# Trends in the Prevalence of Psychological Distress Over Time: Comparing Results From Longitudinal and Repeated Cross-Sectional Surveys

**DOI:** 10.3389/fpsyt.2020.595696

**Published:** 2020-11-26

**Authors:** Peter Butterworth, Nicole Watson, Mark Wooden

**Affiliations:** ^1^Melbourne Institute: Applied Economic and Social Research, The University of Melbourne, Parkville, VIC, Australia; ^2^Research School of Population Health, The Australia National University, Canberra, ACT, Australia

**Keywords:** psychological distress, mental health, prevalence, epidemiology, longitudinal, Australia

## Abstract

**Background:** While there is discussion of increasing rates of mental disorders, epidemiological research finds little evidence of change over time. This research generally compares cross-sectional surveys conducted at different times. Declining response rates to representative surveys may mask increases in mental disorders and psychological distress.

**Methods:** Analysis of data from two large nationally representative surveys: repeated cross-sectional data from the Australian National Health Survey (NHS) series (2001–2017), and longitudinal data (2007–2017) from the Household, Income and Labor Dynamics in Australia (HILDA) Survey. Data from each source was used to generate weighted national estimates of the prevalence of very high psychological distress using the Kessler Psychological Distress scale (K10).

**Results:** Estimates of the prevalence of very high psychological distress from the NHS were stable between 2001 and 2014, with a modest increase in 2017. In contrast, the HILDA Survey data demonstrated an increasing trend over time, with the prevalence of very high distress rising from 4.8% in 2007 to 7.4% in 2017. This increase was present for both men and women, and was evident for younger and middle aged adults but not those aged 65 years or older. Sensitivity analyses showed that this increase was notable in the upper end of the K10 distribution.

**Conclusions:** Using household panel data breaks the nexus between declining survey participation rates and time, and suggests the prevalence of very high psychological distress is increasing. The study identifies potential challenges in estimating trends in population mental health using repeated cross-sectional survey data.

## Introduction

Mental disorders are a leading cause of disability worldwide (1). In Australia, approximately 20% of adults experience a mental disorder each year (2). Both lay and professional literatures claim there is an increasing prevalence of mental disorders, with many references to an epidemic of mental illness. For example, the Australian National Mental Health Commission [(3), page 14] reported that “we are facing a mental ill-health epidemic which is causing needless suffering, crises and premature deaths.” The research evidence to back such claims is, however, less clear.

The strongest research evidence examining the prevalence of mental disorders over time comes from a systematic review of community-based representative studies that assessed (any) anxiety disorder or major depressive disorder meeting the clinical threshold according to either DSM or ICD criteria (4). The review considered 95 studies assessing anxiety disorders and 144 studies examining major depressive disorder conducted between 1990 and 2010 and found no evidence of an increase in either anxiety or major depressive disorders over the study period. Evidence showing an increase in the number of anxiety or depression cases was a reflection of population growth or changing population age profiles.

A major challenge for studies seeking to evaluate secular trends in the prevalence of mental disorders is accounting for the concurrent change in the diagnostic criteria of mental disorders and change in the research instruments used (5). Some studies overcome these concerns by analyzing repeated cross-sectional or longitudinal surveys that use consistent measures over time (6). A recent meta-analysis of repeated cross-sectional studies that examined a range of measures (clinical interviews, symptom and distress scales) found a relatively small increase in prevalence over time and also concluded this likely reflected socio-demographic change (7). The Canadian Community Health Survey and the (Canadian) National Population Health Survey used the same version of the Composite International Diagnostic Interview Short Form for Major Depression (8, 9) over time and showed stability in the prevalence of Major Depressive Episodes (MDE). Similarly, a number of studies have analyzed time series data from the United States' National Survey on Drug Use and Health (NSDUH) which has used the same measure of MDE. Mojtabai and Jorm (10) analyzed this data and reported stability in MDE over time. However, Weinberger et al. (11, 12) and Twenge et al. (12) also analyzed data from the NSDUH and reported increasing prevalence. This difference may reflect that the recent studies evaluated change over a longer follow-up period and including younger respondents (aged 12–17 years) in whom the time trend was strongest.

Other studies have investigated change over time in population mental health using general mental health scales such as the Kessler Psychological Distress scale [K10; (13)]. While the K10 is not a diagnostic instrument, the probability is high that those who score above the cut-point for very high distress have a common mental disorder [i.e., over 85%; (14)]. Analysis of repeated cross-sectional survey data from Australia (5, 15), Canada (9) and the United States (10) have shown stability in the prevalence of very high distress on the K10 over time. However, Twenge et al.'s (12) analysis using the shorter K6 did find evidence of increasing distress, particularly from 2013 onwards and among younger respondents [c.f. (16)].

Analysis of other indicators of mental health are also argued to provide evidence of an increase over time in mental disorders. Stephenson et al. (17) reported a 60% increase in psychotropic drug use in Australia (including a 95% increase in antidepressant medication use) over the period from 2000 to 2011 [see also (10)]. However, analysis of person-level data suggests this may reflect increasing treatment duration and dosage in the context of declining incidence [e.g., (18, 19)]. Similarly, other analysis has shown increases over time in self-reported mental health service use (10, 20, 21) and in self-reported diagnosis with a mental disorder (9). This may be a consequence of changes in diagnostic practices, improvement in community mental health literacy, or greater help-seeking behavior that results in an increased likelihood of diagnosis and treatment among those experiencing mental disorders (9, 22).

Given most of the research examining trends in the prevalence of mental health over time is from repeated cross-sectional studies, findings may also be influenced by (reverse) confounding due to a systematic decline in survey response rates over time (23). The response rate to the Australian National Health Survey (NHS) has declined from 92% in 2001 to 76.1% for the 2017/18 survey. Survey non-response is typically greatest among the young, those in poorer socioeconomic circumstances and those with poor health (24). Thus, over time, a decline in survey response rates may increasingly under-represent those with mental disorders.

To provide a different perspective on this issue, the current study will assess trends in very high psychological distress over time using longitudinal data from a large nationally representative household panel study: the Household, Income and Labor Dynamics in Australia (HILDA) Survey. The HILDA Survey commenced in 2001 and involves annual interviews with around 14,000 participants. The HILDA Survey is based on a multi-stage area sample of households, and seeks to interview all household members aged 15 years or older. The HILDA Survey is a high quality survey that has been the basis of over 1,250 peer-reviewed academic journal articles and over 450 reports, chapters and books^1^.

There are several features of the HILDA Survey that help avoid the limitations of repeated cross-sectional survey data. The HILDA Survey assesses the same individuals repeatedly over time. Therefore, each individual serves as their own control in estimating time trends in the prevalence of very high psychological distress. This approach separates the assessment of distress at each wave from the recruitment of participants (and therefore from the systematic decline in survey response rates). The K10 was introduced into the HILDA Survey at wave seven and, therefore, this analysis excludes the early waves of the study when non-response was highest (25). We also expect greater accuracy in participants' responses reflecting their household's long-term engagement in the study, and that the inclusion of the K10 in a self-complete module (rather than assessed in personal interview as the NHS) may increase participants' willingness to report their mental health symptoms (26).

The aim of this study, therefore, is to compare estimates of the prevalence of very high psychological distress in the Australian population between 2007 and 2017 using data from the repeated cross-sectional NHS series and the longitudinal HILDA Survey. We also use HILDA Survey data to examine age and gender differences over time.

## Methods

### Data

The primary data source is the Household, Income and Labor Dynamics in Australia (HILDA) Survey, an indefinite-life household panel survey (25). The study commenced in 2001 with a representative sample of Australian households residing in private dwellings. Annual interviews are conducted with household members aged 15 years or older. The initial sample of households (response rate = 66%) generated a sample of 13,969 persons (from 15,127 eligible persons). Annual re-interview rates have been high, rising from 87% in wave two to over 94% in wave five, and over 96% from wave nine onwards. The sample of HILDA Survey participants interviewed at each wave is dynamic: it is subject to attrition but also grows as children in the household reach the age of 15 years and when new adults join the original households, and it follows original members as they move into new households. A population-wide sample refreshment was undertaken in wave 11, however to achieve the benefits described above, this new cohort is not included in our analytical sample. The HILDA Survey was approved by the Human Research Ethics Committee at the University of Melbourne.

The outcome variable for this analysis is derived from the 10-item Kessler Psychological Distress Scale (K10) which was introduced into the HILDA Survey in wave seven (2007) and is included in every second wave (i.e., biennial). While the principal mode of data collection for the HILDA Survey is a structured face-to-face interview, some measures, including the K10, are included in a separate self-complete paper questionnaire (SCQ). This questionnaire is associated with additional non-response—the annual return rate for the SCQ has averaged 89.8%. To enable direct comparability with the NHS, the analysis sample is further restricted to persons aged 18 years or older. Thus, the dataset used in this analysis comprises 67,596 observations from 17,572 persons at (up to) six different time points. The biennial response rate (the proportion of in-scope respondents reinterviewed 2 years later) in this sample averaged 94.0%.

The paper reports comparison with published estimates from the various rounds of the National Health Surveys conducted by the Australian Bureau of Statistics (ABS) in 2001, 2004/05, 2007/08, 2011/12, 2014/15, and 2017/18. These are cross-sectional surveys that sample private dwellings and select one adult (18 years or older) and, where possible, one child for interview. Sample sizes have ranged from 15,800 to 21,800 dwellings, and household participation rates have declined from 92% in 2001 to 76% in 2017/18. The NHS methodology is explained in detail on the ABS website (27–32).

### Measures

The K10 scale measures the experience of non-specific psychological distress over the past 4 weeks. Following Andrews and Slade (14), responses to the K10 items were summed to produce a scale ranging from 10 to 50. The K10 was designed to have optimal sensitivity in the part of the population distribution where serious mental health disorders are most common (13). It is highly skewed, with over 56% of the current HILDA sample scoring between 10 and 14. The K10 is an effective screener for mental health disorders within the population (33, 34) and this analysis focuses on those identified at “very high risk” of psychological distress [i.e., scores of 30–50; (35)].

Multivariate models included relevant covariates: age (modeled as three categories–young (aged 18–34 years), middle-aged (aged 35–65 years)) and old (65 years and older), gender, equivalised disposable household income (in previous financial year) categorized into quintiles, housing type (separate house, unit/apartment, or non-private dwelling), and sample status (permanent or temporary sample member). Time is represented by a series of binary variables indicating year of response or as a continuous measure representing study year.

### Statistical Analysis

Analyses were conducted using Stata 15 (36). The NHS data were subject to a test for trend over time in proportions (based on weighted sample size). For analysis of the HILDA Survey, estimates and logistic models were based on data weighted to adjust for selection, non-response (including non-response to the SCQ), and sample clustering and stratification using the Stata svy commands. The initial simple models assessed linear trends over time for the total population, as well as for subpopulations based on gender and age group, using adjusted Wald tests (based on an approximate *F* test). A set of sensitivity tests investigated the robustness of the findings. These involved analyses: restricted to participants with data from at least half of all waves; restricted to permanent panel members (i.e., excluding those who temporarily join a participating household); using different modeling approaches (linear regression on K10 scale score; quantile regression); and examining the effect of applying different cut-points on the K10 scale.

## Results

**Figure 1:**
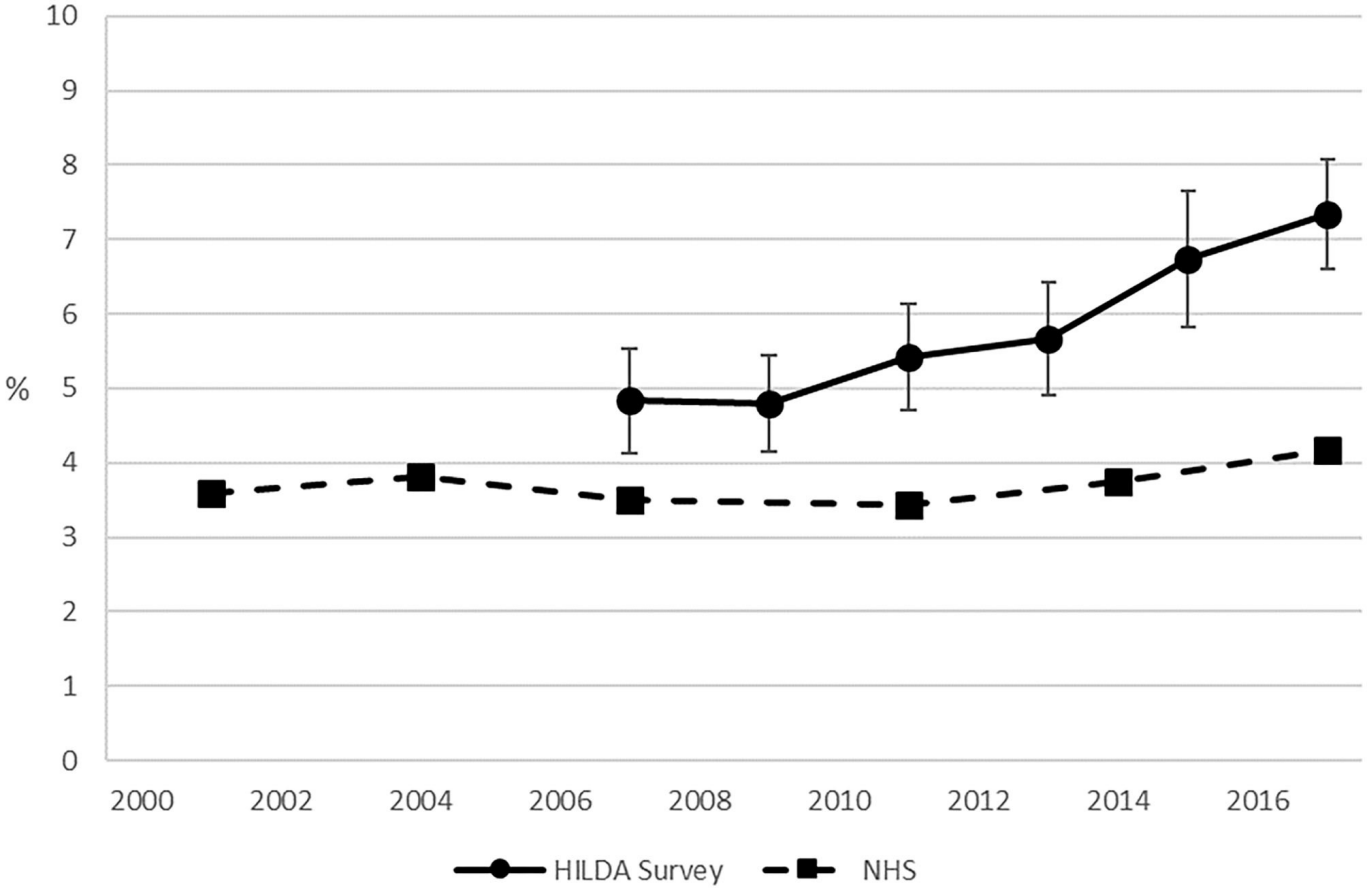
The prevalence of persons in the Australian adult population (18 years +) at “very high” risk of psychological distress: The HILDA Survey and National Health Surveys compared. Notes: The NHS observations for 2004, 2007, 2011, and 2014 were actually collected over the 12-month periods 2004/05, 2007/08, 2011/12, and 2014/15, respectively. The HILDA Survey sample excludes the refreshment sample added in 2011. Sources: HILDA Survey—Department of Social Services / Melbourne Institute of Applied Economic and Social Research (2018); NHS—ABS (2002, 2006, 2009, 2012, 2015).

Figure 1 presents estimates of the prevalence of Australian adults at very high risk of psychological distress from the HILDA Survey and the various rounds of the NHS. The NHS data, which span the period 2001 to 2017/18, showed modest evidence of an increasing trend (χ2 = 6.47, df = 1, *p* = 0.011). Exclusion of the outlying datapoint from 2017 (4.2%) showed the trend from 2001 to 2014/15 was not significant (χ2 = 0.04, df = 1, *p* = 0.84) with prevalence rates in a narrow range between 3.4 and 3.8%. The estimates from the HILDA Survey, which span the period from 2007 to 2017, are consistently higher than those from the NHS and exhibit an upward (linear) trend (F1,391 = 43.00, *p* < 0.001), rising from around 4.8% (95% confidence interval [CI] = 4.1–5.5) in 2007 to 7.4% (CI = 6.7–8.1) in 2017. The exclusion of 2017 did not eliminate the linear effect (F1,391 = 19.48, *p* < 0.001).

### Detailed Analysis of HILDA Survey Data

Overall, the prevalence of very high psychological distress was greater for women than men (6.6% vs. 5.0%; *p* < 0.001) and both men and women showed an increase in the risk of very high psychological distress over time (men: F1,390 = 25.46, *p* < 0.001; women: F1,391 = 20.49, *p* < 0.001), though the pattern was not strictly linear and differed by gender (see Supplementary Figure 1). For men, the greatest wave-to-wave increase in the prevalence of very high distress occurred between waves 9 and 11 (*p* = 0.025) and between waves 13 and 15 (*p* = 0.054). For women the greatest increase occurred between waves 13 and 15 (*p* = 0.077) and waves 15 and 17 (*p* = 0.025).

Estimates of very high psychological distress for the three age groups are presented in Table 1. Analysis showed a significant interaction between age group and (continuous) time (F2,390 = 6.48, *p* < 0.001). The increasing prevalence of very high psychological distress over time was evident in the youngest (F1,291 = 33.6, *p* < 0.001) and, to a lesser extent, the middle-aged (F1,391 = 18.33, *p* < 0.001) but not the oldest group of respondents (F1,391 = 0.18, *p* = 0.67).

**Table 1:** The prevalence of persons in the Australian adult population at “very high” risk of psychological distress by age (%), 2007–2017 (HILDA Survey).

**Age group (years)**	**2007**	**2009**	**2011**	**2013**	**2015**	**2017**	P^*^
18–34	5.8	5.9	6.3	7.6	8.3	10.2	*p* < 0.001
	(4.7, 7.0)	(4.8, 7.1)	(5.0, 7.6)	(6.2, 9.0)	(6.8, 9.8)	(8.7, 11.7)	
35–64	4.9	4.6	5.4	5.3	6.9	7.5	*p* < 0.001
	(3.8, 5.9)	(3.7, 5.6)	(4.5, 6.3)	(4.4, 6.2)	(5.5, 8.2)	(6.3, 8.6)	
65+	2.7	3.1	3.9	3.2	3.7	2.3	*p* = 0.674
	(1.7, 3.7)	(2.1, 4.1)	(1.7, 6.1)	(1.9, 4.5)	(2.5, 4.9)	(1.5, 3.1)	
Total (18+)	4.8	4.8	5.4	5.7	6.8	7.4	*p* < 0.001
	(4.2, 5.6)	(4.2, 5.5)	(4.7, 6.2)	(4.9, 6.5)	(5.9, 7.7)	(6.7, 8.1)	

*Notes: 95% confidence intervals in parentheses*.

* *Wald test for trend*.

*Source: Department of Social Services/Melbourne Institute of Applied Economic and Social Research (2018)*.

Summary results from the final models, focusing on time, are presented in Table 2. The initial model (A) included only the effect of time and shows that the odds of very high psychological distress increased over time. Analysis of continuous linear time produced an Odds Ratio of 1.05 (CI = 1.03–1.07), indicating that each additional year increased the odds of very high psychological distress by 5%. The second model (B) included all covariates and the Odds Ratios were effectively unchanged, with increasing odds of very high psychological distress over time. Considering age as a continuous variable and rescaled to represent a 5-year interval, an Odds Ratio of 0.90 (CI = 0.88–0.92) indicated that, for each additional 5 years of age, an individual's odds of reporting very high psychological distress were 10% lower. The complete model results, including all covariates and reporting age in years, is in Supplementary Table 1. There was a trend of decreasing rates of very high distress with increasing age, women had higher odds than men (OR = 1.30; CI = 1.13–1.48), temporary household members had greater risk of psychological distress than permanent household members (OR = 1.32; CI = 1.12–1.56), and there was an inverse gradient by household income quintile. Overall, housing type was not independently associated with very high psychological distress.

**Table 2:** Odds ratios from key logit regressions of the likelihood of being at “very high” risk of psychological distress.

	**Model A Simple**	**Model BFull multivariate model**	**Model C Participating in at least 50% of waves**	**Model DPermanent sample members only**
A) Discrete time (year): 2007 (ref)	1.00	1.00	1.00	1.00
2009	0.99	0.99	0.93	0.97
	(0.84, 1.18)	(0.83, 1.18)	(0.72, 1.08)	(0.81, 1.17)
2011	1.13	1.13	1.16	1.14
	(0.94, 1.35)	(0.94,1.35)	(0.99, 1.37)	(0.94, 1.38)
2013	1.18	1.19	1.16	1.15
	(1.01, 1.39)	(1.01, 1.40)	(1.00, 1.36)	(0.97, 1.36)
2015	1.43	1.42	1.41	1.40
	(1.19, 1.71)	(1.18, 1.72)	(1.18, 1.69)	(1.16, 1.70)
2017	1.57	1.57	1.47	1.57
	(1.34, 1.83)	(1.33, 1.84)	(1.25, 1.75)	(1.33, 1.86)
B) Continuous (linear) time	1.05	1.05	1.05	1.05
	(1.03, 1.07)	(1.04, 1.07)	(1.03, 1.07)	(1.03, 1.07)

*Notes: 95% confidence intervals in parentheses*.

*Source: Department of Social Services/Melbourne Institute of Applied Economic and Social Research (2018)*.

### Sensitivity Analyses

A number of analyses tested the robustness of the main findings. Restricting the main analysis to individuals with data from at least three waves (Model C, Table 2) or to permanent sample members (Model D, Table 2) had very little impact on the estimated coefficients.

Alternative modeling approaches are presented in Table 3. There was a small but significant effect of time in Ordinary Least Squares (OLS) regression models (A) on the K10 scale scores (a 0.07-point increase each year; 0.63 difference between waves seven and 17). The quantile regression models (B) present the wave seven scores corresponding to the 25th, median, 75th, 85th, 90th, 95th, and 97th percentiles, and the difference at each later wave. There was no change over time in the lower quintiles (e.g., the median K10 score was 15 at all waves). At higher quintiles the K10 scores increased over time: the scores for the top three percent of Australian adults increased from 31 at wave seven to 34 at wave 15. The lack of differentiation at lower K10 scores may reflect the skewed K10 distribution, with most respondents reporting no/low symptoms. However, a plot contrasting the inverse cumulative distribution of K10 scores at waves seven and 17 (Supplementary Figure 2) shows a higher proportion of wave 17 respondents identified at most points of the K10 scale, not just the established cut point. Using each K10 score as a cut-point, Supplementary Figure 3 shows that a significantly greater proportion of respondents are identified at wave 17 than wave seven at all K10 scores between 15 and 40, with the absolute difference most pronounced at mid-range K10 scores between 15 and 29. Finally, the relative difference between waves at different cut-points increased as the cut-point became stricter (and as the proportion of respondents identified became smaller; see Table 3C).

**Table 3:** Alternative regression models of psychological distress on (categorical and linear) time: OLS regression, quantile regression at the 25th, 50th, 75th, 85th, 90th, 95th, and 97th percentile, and logit models using different cut-points on the K10, 2007–2017 (HILDA Survey).

	**Categorical time**						***Overall***
	**2007 (Reference category: mean or quantile)**	**2009**	**2011**	**2013**	**2015**	**2017**	
A. OLS regression (mean)							
	Ref 15.91	−0.08 (−0.28–0.12)	0.04 (−0.18–0.27)	0.07 (−0.13–0.28)	**0.39 (0.15–0.63)**	**0.63** **(0.38−0.89)**	**0.07 (0.04–0.09)**
B. Quantile regression (score)							
25th percentile	Ref 11	0 (−0.12–0.12)	0 (−0.12–0.12)	0 (−0.12–0.12)	0 (−0.12–0.12)	0 (−0.12–0.12)	0 (0.00–0.00)
Median	Ref 15	0 (−0.20–0.20)	0 (−0.19–0.19)	0 (−0.19–0.19)	0 (−0.19–0.19)	0 (−0.19–0.19)	0 (−0.02–0.02)
75th percentile	Ref 18	0 (−0.48–0.48)	0 (−0.48–0.48)	0 (−0.48–0.48)	**1** **(0.24–1.76)**	**1** **(0.52–1.48)**	**0.13 (0.08–0.17)**
85th percentile	Ref 22	0 (−0.42–0.42)	**1** **(0.58–1.42)**	**1** **(0.58–1.42)**	**1** **(0.58–1.42)**	**2** **(1.59–2.41)**	**0.17 (0.11–0.22)**
90th percentile	Ref 25	0 (−0.65–0.65)	0 (−0.64–0.64)	0 (−0.64–0.64)	**1** **(0.35–1.65)**	**2** **(1.00–3.00)**	**0.3 (0.22–0.38)**
95th percentile	Ref 29	0 (−0.66–0.66)	0 (−0.66–0.66)	**1** **(0.35–1.65)**	**1** **(0.35–1.65)**	**2** **(1.36–2.64)**	**0.2 (0.14–0.36)**
97th percentile	Ref 31	1 (−0.35–2.35)	**2** **(1.15–2.85)**	**2** **(0.69–3.31)**	**3** **(1.65–4.35)**	**3** **(1.67–4.33)**	**0.25 (0.15–0.35)**
C. Logistic regression (Odds Ratios) with different cut-points on K10						
Cut-point = 33	Ref 1.00	1.10 (0.88–1.39)	**1.24 (1.02–1.52)**	**1.28** **(1.05–1.56)**	**1.50 (1.22–1.84)**	**1.65** **(1.34–2.02)**	**1.05 (1.03–1.07)**
Cut-point = 32	Ref 1.00	1.06 (0.86–1.31)	**1.24 (1.02–1.52)**	**1.24** **(1.04–1.48)**	**1.47 (1.20–1.81)**	**1.59** **(1.31–1.94)**	**1.05 (1.03–1.07)**
Cut-point = 31	Ref 1.00	1.00 (0.83–1.21)	**1.20 (1.00–1.44)**	**1.23** **(1.04–1.46)**	**1.43 (1.18–1.72)**	**1.61** **(1.36–1.91)**	**1.05 (1.04–1.07)**
Cut-point = 30 (standard)	Ref 1.00	0.99 (0.74–1.18)	1.13 (0.94–1.35)	**1.18** **(1.01–1.39)**	**1.43 (1.19–1.71)**	**1.57** **(1.34–1.83)**	**1.05 (1.04–1.07)**
Cut-point = 29	Ref 1.00	0.97 (0.82–1.13)	1.12 (0.95–1.31)	1.13 (0.98–1.32)	**1.35 (1.14–1.59)**	**1.49** **(1.28–1.74)**	**1.05 (1.03–1.06)**
Cut-point = 28	Ref 1.00	0.95 (0.82–1.09)	1.09 (0.94–1.27)	1.13 (0.98–1.29)	**1.32 (1.13–1.54)**	**1.43** **(1.23–1.65)**	**1.04 (1.03–1.06)**
Cut-point = 27	Ref 1.00	0.97 (0.85–1.10)	1.11 (0.96–1.28)	**1.16** **(1.03–1.31)**	**1.32 (1.15–1.52)**	**1.44** **(1.25–1.65)**	**1.04 (1.03–1.06)**

* *Reference category represents mean in 2007 for OLS regression model, relevant quintile in 2007 for quintile regression models, and 2007 Odds Ratio reference for logistic regression models. Bold indicates significant at p < 0.05*.

## Discussion

The existing epidemiological literature provides little evidence that the prevalence of mental disorders or very high psychological distress has increased over time. For example, previous analysis of the NHS data showed that the prevalence of very high psychological distress in Australia was stable between 2001 and 2014 (5). However, our analysis did find an increase in very high distress in the most recent (2017/18) NHS data [see also (15)]. In contrast, in the HILDA Survey data the prevalence of very high psychological distress was consistently greater than the NHS estimates and, more critically, showed that rates had risen steeply over time: from 4.8% of the adult population in 2007 to 7.3% by 2017. This pattern of increasing rates of very high psychological distress was found for both men and women and the trends were strongest for younger respondents and most evident in recent years (11, 12).

In examining the robustness of the results we found only a modest increase in mean K10 scores across waves, with the quintile analysis suggesting the increase in psychological distress was largely restricted to the high K10 scores. When we more closely examined the distribution of scores, we found evidence of an increase at all but the lowest points on the K10 scale over time. Nevertheless, most of the increase occurs in the upper half of the distribution. This is consistent with the nature of the K10 scale, which was developed to be sensitive at the extreme range of the distribution of distress and to differentiate between those with and without serious mental illness (34). These sensitivity analyses support our main finding of a significant increase in rates of very high psychological distress in Australia over the past decade. While examination of the causes of the increase in distress over time is beyond the scope of this study, possible explanations canvassed in the literature include the growing ubiquity of social media and electronic communication (12), loneliness (37) and perceptions of job insecurity since the Global Financial Crisis/Great Recession in 2007–2009 (38).

There were several reasons why we posited the HILDA Survey may provide a more accurate indication of trends in population mental health over time than other data sources such as the NHS series. Our primary concern was about the impact of declining survey participation rates over time on NHS estimates (24). Survey non-response is greatest among the most vulnerable members of society, including the young, the poor and those with poor health. These are the individuals most likely to experience poor mental health. Declining survey participation rates are particularly problematic when using repeated cross-sectional surveys to assess trends over time as change in response rate systematically co-varies with time. If cross-sectional samples increasingly underrepresent those with mental ill-health, it introduces a reverse confound that could mask (or even reverse) any secular increase in population levels of very high psychological distress. The use of longitudinal data, following the same households (and largely the same individuals), breaks this nexus between sample recruitment and time. We acknowledge that attrition affects longitudinal surveys, but the likelihood of panel attrition declines with increasing time in study. As the K10 was first included in the HILDA Survey in wave seven, the current analysis excludes the early waves of the study when non-response was highest.

The HILDA Survey methodology is also likely to generate a more inclusive sample. The approach of interviewing all household members aged 15 years or older will encourage engagement by individuals who, in traditional single person studies, may not participate. The longitudinal design also means individuals who decline to participate at one point may return in subsequent waves: in our analysis 21.7% of individuals who did not participate at one wave returned in a subsequent wave.

We also anticipated that the K10 data in the HILDA Survey may be more accurate than that from the NHS. Wooden (26) discussed how social desirability may influence responses to questions about psychological distress [see also (39)], with under-reporting more pronounced in the presence of others. Thus, K10 data collected through personal interviews (such as in the NHS) may be subject to greater under-reporting than self-administered methods (as used in the HILDA Survey). In addition, given their long-term study engagement (for up to 17 years), HILDA Survey participants are likely to have higher levels of trust and confidence in the interviewers (most of whom are allocated the same households each year), and greater commitment to the study. The panel conditioning associated with long-term participation in the survey (40, 41) may have also increased the accuracy of survey responses on sensitive topics.

There are a number of limitations that must be acknowledged. First, the analysis is restricted to Australian data and the findings may not generalize to other countries. Second, the HILDA Survey is also subject to non-response at commencement and attrition over time. The dataset includes weights designed to adjust for non-response and attrition to ensure estimates more closely resemble the Australian population (42). However, weighting is an inexact science and cannot address unmeasured sources of non-response (23). Nonetheless, poor mental health is associated with elevated rates of attrition in the HILDA Survey (43), and this would result in an underestimate of the rate of very high psychological distress.

Third, the focus on a cohort of households/individuals originally recruited in 2001 means the HILDA Survey sample may not reflect recent changes in the Australian population. Between 2006 and 2016, the proportion of overseas born Australian residents increased from 24.6% to 28.5% (44). The “healthy immigrant effect” (45) shows that immigrants (excluding refugees) generally have better health than native-born residents. However, it seems implausible that the absence of recently arrived foreign-born immigrants in the HILDA sample (< 4% of the total Australian population) could account for a 2.6% increase in rates of very high psychological distress.

We acknowledge that our hypothesis that declining survey response rates in the NHS may mask a real-world increase in very high psychological distress is not directly tested. Keyes and colleagues (23) showed the health of survey participants was better than that of the US population when assessed using an objective marker (mortality). However, they did not find that this inaccuracy increased over time, as would be expected with declining survey response rates. There is no readily identifiable gold standard against which to evaluate survey reports of mental health.

While we argued longitudinal data enabled us to demonstrate evidence of the increasing prevalence of mental ill-health over time, other longitudinal studies have not shown this pattern of results. Simpson and colleagues (9) analyzed the longitudinal Canadian National Population Health Survey and reported stability in very high psychological distress over time. However, they examined an earlier period (1994–2008) and the increase in the prevalence of mental disorders/distress may be a more recent phenomenon. Conversely, some studies reporting analysis of repeated cross-sectional data (11, 12) have also described a recent increase in rates of mental disorders. The data used in these analyses (the NSDUH) showed a more modest decline in survey response rates [from 74.2% to 67.1% between 2008 and 2017; (46)].

## Conclusions

Our analysis of nationally representative longitudinal data from the HILDA Survey showed that the prevalence of very high psychological distress has increased markedly over the past decade, with much of this increased burden falling upon younger Australians. The generalizability and clinical implications of this finding warrant further investigation. The HILDA Survey, given its emphasis on measuring economic, social and family circumstances, provides an important resource for future research to investigate how broader social and economic circumstances may have increased the burden of mental ill-health within the Australian community. Representative cross-sectional surveys (such as the NHS) will continue to have a key role in health monitoring, but declining survey participation rates may require a reconsideration of how best to assess and monitor trends in population mental health.

## Data Availability Statement

Publicly available datasets were analyzed in this study. This data can be found here: The Australian Data Archive (see https://dataverse.ada.edu.au).

## Ethics Statement

The studies involving human participants were reviewed and approved by Human Research Ethics Committee at the University of Melbourne. Written informed consent for participation was not required for this study in accordance with the national legislation and the institutional requirements.

## Author Contributions

MW contributed to the design of the study. NW and PB contributed to data analysis. All authors contributed to interpretation of data, drafting and revising of the manuscript, and approved the final manuscript.

## Conflict of Interest

The authors declare that the research was conducted in the absence of any commercial or financial relationships that could be construed as a potential conflict of interest.

## Footnotes

^1^See https://melbourneinstitute.unimelb.edu.au/hilda/publications
