# Supplementary material for: Trends in the Prevalence of Psychological Distress Over Time: Comparing Results From Longitudinal and Repeated Cross-Sectional Surveys
Source: Front Psychiatry. 2020 Nov 26;11:595696. doi: 10.3389/fpsyt.2020.595696 (PMC7725766; doi:10.3389/fpsyt.2020.595696)
Supplement: Supplementary file 1 [file Data_Sheet_1.docx]

Supplementary Material

# Supplementary Figures and Tables

## Supplementary Table 1. Odds ratios and 95% Confidence Intervals from final multivariate logit regression models assessing the odds of being at “very high” risk of psychological distress, reporting both discrete time (Model A) and continuous/linear time (Model B).

|  | **A: Discrete time** | | **B: Continuous time** | |
| --- | --- | --- | --- | --- |
|  | OR | 95% CI | OR | 95% CI |
| **Study year: 2007 (ref)** | 1.00 |  |  |  |
| 2009 | 0.98 | (0.82-1.17) |  |  |
| 2011 | 1.13 | (0.94-1.35) |  |  |
| 2013 | 1.19 | (1.01-1.40) |  |  |
| 2015 | 1.42 | (1.17-1.72) |  |  |
| 2017 | 1.57 | (1.33-1.84) |  |  |
| **Continuous (linear) time** |  |  | 1.05 | (1.04-1.07) |
| **Age in years: 18 (ref)** | 1.00 |  |  |  |
| 19 | 0.89 | (0.61-1.32) |  |  |
| 20 | 1.24 | (0.89-1.72) |  |  |
| 21 | 0.92 | (0.61-1.40) |  |  |
| 22 | 1.23 | (0.84-1.80) |  |  |
| 23 | 1.02 | (0.65-1.60) |  |  |
| 24 | 1.51 | (1.02-2.24) |  |  |
| 25 | 1.27 | (0.81-1.98) |  |  |
| 26 | 0.75 | (0.49-1.17) |  |  |
| 27 | 1.14 | (0.75-1.74) |  |  |
| 28 | 1.16 | (0.72-1.88) |  |  |
| 29 | 0.86 | (0.55-1.35) |  |  |
| 30 | 1.05 | (0.66-1.65) |  |  |
| 31 | 0.72 | (0.45-1.14) |  |  |
| 32 | 1.01 | (0.63-1.62) |  |  |
| 33 | 1.07 | (0.69-1.67) |  |  |
| 34 | 0.94 | (0.59-1.48) |  |  |
| 35 | 0.86 | (0.53-1.38) |  |  |
| 36 | 0.74 | (0.41-1.32) |  |  |
| 37 | 0.91 | (0.59-1.39) |  |  |
| 38 | 0.86 | (0.54-1.37) |  |  |
| 39 | 0.61 | (0.40-0.93) |  |  |
| 40 | 0.76 | (0.50-1.18) |  |  |
| 41 | 0.84 | (0.55-1.28) |  |  |
| 42 | 0.71 | (0.46-1.09) |  |  |
| 43 | 0.87 | (0.57-1.32) |  |  |
| 44 | 0.71 | (0.46-1.09) |  |  |
| 45 | 0.79 | (0.54-1.16) |  |  |
| 46 | 0.70 | (0.46-1.08) |  |  |
| 47 | 0.89 | (0.56-1.40) |  |  |
| 48 | 0.92 | (0.59-1.44) |  |  |
| 49 | 0.85 | (0.57-1.27) |  |  |
| 50 | 0.53 | (0.34-0.82) |  |  |
| 51 | 0.88 | (0.55-1.40) |  |  |
| 52 | 0.55 | (0.34-0.89) |  |  |
| 53 | 0.99 | (0.58-1.71) |  |  |
| 54 | 0.93 | (0.58-1.50) |  |  |
| 55 | 0.51 | (0.32-0.83) |  |  |
| 56 | 0.82 | (0.48-1.41) |  |  |
| 57 | 1.23 | (0.72-2.10) |  |  |
| 58 | 0.75 | (0.45-1.26) |  |  |
| 59 | 0.68 | (0.42-1.10) |  |  |
| 60 | 0.73 | (0.38-1.43) |  |  |
| 61 | 0.59 | (0.32-1.07) |  |  |
| 62 | 0.86 | (0.50-1.49) |  |  |
| 63 | 0.64 | (0.33-1.21) |  |  |
| 64 | 0.67 | (0.31-1.43) |  |  |
| 65 | 0.31 | (0.15-0.64) |  |  |
| 66 | 0.21 | (0.12-0.37) |  |  |
| 67 | 0.24 | (0.13-0.42) |  |  |
| 68 | 0.27 | (0.14-0.50) |  |  |
| 69 | 0.38 | (0.20-0.72) |  |  |
| 70 | 0.31 | (0.17-0.58) |  |  |
| 71 | 0.35 | (0.18-0.69) |  |  |
| 72 | 0.22 | (0.12-0.39) |  |  |
| 73 | 0.23 | (0.12-0.45) |  |  |
| 74 | 0.19 | (0.10-0.35) |  |  |
| 75 | 0.20 | (0.06-0.67) |  |  |
| 76 | 0.21 | (0.11-0.37) |  |  |
| 77 | 0.12 | (0.06-0.25) |  |  |
| 78 | 0.33 | (0.16-0.69) |  |  |
| 79 | 0.24 | (0.11-0.52) |  |  |
| 80 | 0.21 | (0.08-0.53) |  |  |
| 81 | 0.13 | (0.06-0.31) |  |  |
| 82 | 0.08 | (0.02-0.28) |  |  |
| 83 | 0.14 | (0.05-0.34) |  |  |
| 84 | 0.23 | (0.09-0.59) |  |  |
| 85 | 0.11 | (0.03-0.46) |  |  |
| 86 | 0.14 | (0.05-0.41) |  |  |
| 87 | 0.18 | (0.08-0.41) |  |  |
| 88 | 0.32 | (0.12-0.88) |  |  |
| 89 | 0.88 | (0.23-3.37) |  |  |
| 90 | 0.20 | (0.04-1.06) |  |  |
| 91 and older | 0.79 | (0.23-2.76) |  |  |
| **Female** | 1.30 | (1.13-1.48) | 1.30 | (1.13-1.48) |
| **Temporary sample member** | 1.32 | (1.12-1.56) | 1.33 | (1.12-1.56) |
| **Quintiles of equivalized disposable income: Lowest (1; ref)** | 1.00 |  | 1.00 |  |
| 2nd quintile | 0.49 | (0.40-0.59) | 0.49 | (0.40-0.59) |
| 3rd quintile | 0.38 | (0.31-0.47) | 0.38 | (0.31-0.47) |
| 4th quintile | 0.23 | (0.19-0.29) | 0.23 | (0.19-0.29) |
| 5th (highest) quintile | 0.20 | (0.16-0.25) | 0.20 | (0.16-0.25) |
| **Dwelling type: Separate house (ref)** | 1.00 |  | 1.00 |  |
| Other | 0.31 | (0.04-2.30) | 0.31 | (0.04-2.27) |
| House with attached shop | 1.62 | (0.59-4.46) | 1.62 | (0.59-4.45) |
| Semi-detached, one story | 1.50 | (1.11-2.01) | 1.50 | (1.11-2.01) |
| Semi-detached, 2 or more stories | 1.07 | (0.77-1.50) | 1.07 | (0.77-1.50) |
| Flat/unit/apartment in one-story | 1.48 | (1.06-2.07) | 1.48 | (1.05-2.07) |
| Flat/unit/apartment in two-story. | 0.99 | (0.71-1.39) | 0.99 | (0.71-1.39) |
| Flat/unit/apartment in three story | 0.68 | (0.44-1.07) | 0.68 | (0.44-1.07) |
| Flat/unit/apartment in four to nine stories | 0.74 | (0.46-1.20) | 0.74 | (0.46-1.20) |
| Flat/unit/apartment in ten or more stories | 1.23 | (0.62-2.47) | 1.23 | (0.61-2.46) |
| Flat/unit/apartment attached to house | 1.33 | (0.71-2.46) | 1.33 | (0.72-2.46) |
| Flat/unit/apartment attached to shop | 0.47 | (0.16-1.37) | 0.48 | (0.16-1.38) |
| Caravan/Tent/Cabin/Houseboat | 1.13 | (0.66-1.91) | 1.12 | (0.66-1.89) |
|  |  |  |  |  |

**Supplementary Figure 1.** The prevalence of persons in the Australian adult population (18 years +) at “very high” risk of psychological distress by gender, 2007-2017 (HILDA Survey)





Note: The refreshment sample added in 2011 has been excluded.

Source: Department of Social Services / Melbourne Institute of Applied Economic and Social Research (2018).

**Supplementary Figure 2.** Inverse cumulative proportion of K10 scores (weighted) in the HILDA Survey analysis sample, comparing wave 7 and wave 17.

Note: The refreshment sample added in 2011 has been excluded.

Source: Department of Social Services / Melbourne Institute of Applied Economic and Social Research (2018).

**Supplementary Figure 3.** Absolute differences between wave 17 and 7 in the estimated weighted percent of people classified using all possible cut-points across the full K10 scale, with 95 percent confidence intervals (wave 17 - wave 7)


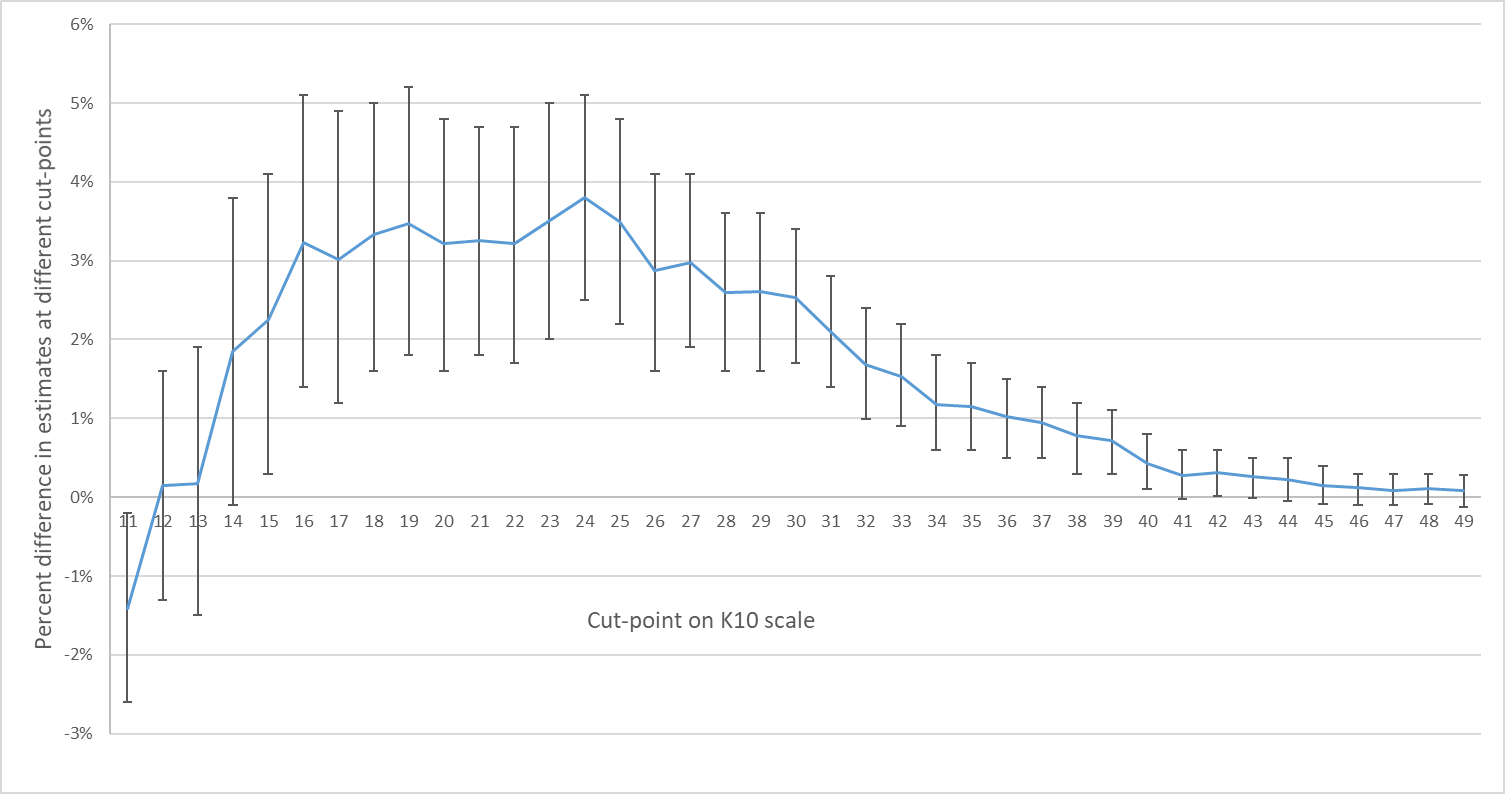


Source: Department of Social Services / Melbourne Institute of Applied Economic and Social Research (2018).
